# Immune Response to *Chlamydophila abortus* POMP91B Protein in the Context of Different Pathogen Associated Molecular Patterns (PAMP); Role of Antigen in the Orientation of Immune Response

**DOI:** 10.3390/toxins1020059

**Published:** 2009-10-13

**Authors:** Vincent Le Moigne, Georges Robreau, Wahib Mahana

**Affiliations:** Unversité de Bretagne Occidentale (UBO), IUT de Quimper, 2, rue de l’Université, 29334 Quimper, France; Email: v.lemoigne@afssa.fr (V.L.M.); robreau@univ-brest.fr (G.R.)

**Keywords:** POMP91B, PAMP, flagellin, immune response, adjuvant

## Abstract

In a previous study, we used bacterial flagellin to deliver antigens such as p27 of *Mycobacterium tuberculosis* to a host immune system and obtained a potent Th1 response compared to those obtained with Freund’s adjuvant and DNA immunization. In the current study, using a POMP91B antigen of *Chlamydophila abortus*, a human and animal pathogen, as a model, we found that this antigen is unable to promote Th1 response. However, this antigen, unlike others, was able to induce a good Th2 response and IL-4 production after immunization by recombinant protein in Freund’s adjuvant or in phosphate buffered saline. Our results suggest that immune response is not only dependent on the immunization adjuvant, but also dependent on the nature of antigen used.

## 1. Introduction

Members of the *Chlamydiaceae* family can cause infections in all animal kingdoms [1]. After the recent reclassification of the *Chlamydiae phylum*, the *Chlamydiaceae* family has been divided into two genus, *Chlamydia* and *Chlamydophila* [2]. The two genus contain nine species and four of them, *Chlamydia trachomatis*, *Chlamydophila pneumoniae*, *Chlamydophila psittaci* and *Chlamydophila abortus*, can cause infections in human [3]. They are Gram-negative bacteria with intracellular growth. Among them, *C. abortus* is identified as the Serovar 1 of *Chlamydia psittaci* [2], which infects epithelial mucosal cells responsible for chlamydioses in a number of animal species.

Infections with *C. abortus* cause abortions in sheep and goats. This bacteria, when transmitted to humans, can equally be responsible for abortions [4,5]. It has been difficult to generate a vaccine against *C. abortus* infection, since this intracellular obligate bacteria is difficult to obtain in large quantities. 

The POMP91B protein belongs to a group of four proteins, named Polymorphic Outer Membrane Proteins (POMPs), of approximately 90 kDa, present in the outer membrane of *C. abortus* [6]. Although they are present in small amounts in the bacteria, these proteins are powerfully immunogenic, as confirmed by serum analysis after abortion in sheep [7], or after injection of live bacteria to mice [8]. POMP91B possesses an *N*-terminal extracellular exposed region [7], responsible for the strong response in sheep which supported its use in an ELISA detection test [9,10]. The structure of this *N*-terminal part is composed of a Beta-barrel [11].

Pathogen Associated Molecular Patterns (PAMPs) are expressed only by micro-organisms and are recognized by eukaryotic cells through the Pattern Recognition Receptors (PRRs) of the innate immune system, such as the Toll-like receptors (TLRs) [12]. The interaction of PAMPs with their corresponding TLRs helps to identify the nature of the PAMP and to guide the adequate adaptive immune response [12]. Muramyl dipeptides, a major element of Freund's complete adjuvant, bacterial DNA, and flagellin are three PAMPs recognized by TLR2, TLR9, and TLR5, respectively. Freund’s adjuvant is usually used with high efficiency to amplify the immune response in experimental animals [13]. DNA technology has been used successfully in the vaccination of animal models against infection with viruses, bacteria, and parasites as well as in anti-tumor therapy, allergies and treatment of autoimmune diseases [14]. *Salmonella* flagellin was also used with partial success as an adjuvant and a carrier for a synthetic peptide representing a neutralizing epitope of influenza virus [15], and to produce antibodies against cholera toxin epitope [16]. The antibody response, systemic (IgG) or secreted (IgA), against flagellin was dependent on the route of immunization [17] and finally, immunization of mice with a fusion protein containing a flagellin-enhanced green protein was capable of stimulating antigen presenting cells and thus develop a specific T-cell response [18]. We previously used this strategy and obtained a good Th1 response against two different bacterial antigens [19,20].

The extracellular localization of the *N*-part of the POMP91B protein [7], as well as the other described POMPs proteins led to the belief that they might play an important immunological role either in diagnosis or in protection. In the present report, we examined the immune response against the *N*-terminal region of the POMP91B protein using different PAMPs as adjuvants and vectors for immunization and compared it with the responses obtained with P27 antigen of tuberculosis. A DNA plasmid encoding this region of POMP91B protein (DNA immunization) was prepared and its immunogenicity was compared with that of the recombinant protein (classical immunization) and with that of a recombinant fusion protein carried by the flagellar filament of bacteria *E. coli* (flagellin immunization). 

Here, we show that the administration of the recombinant POMP91B protein induces the strongest Th2-like response, as indicated by IL-4 and antibody production. In comparison with two other antigens, immunization with the bacteria expressing the partial POMB91B protein into their flagella is not able to induce a strong Th1‑like response, as indicated by the weak cell proliferation and IFN-γ production. The DNA immunization was unable to induce an antigen-specific immune response. 

Regarding the other antigens, the *N*-terminal part of the *C. abortus* POMP91B stimulates a strong Th2 response profile when the immunizations are done with the recombinant protein which does not require Freund’s adjuvant to present a good IL-4 and antibody response. In addition, this antigen is weaker in inducing a Th1 response, regardless of the immunization method used.

## 2. Results

### 2.1. Expression and purification of the N-terminal part of the POMP91B antigen in different systems

**Figure 1:**
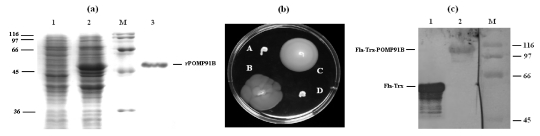
Construction and expression of the recombinant POMP91B protein (a) Coomasie blue staining of the bacterial lysates and purified recombinant *N*-terminal region of POMP91B protein expressed with His-Tag. Lanes 1 and 2: Bacterial extracts of *E. coli* BL21 (DE3) without or with IPTG induction respectively. Lane 3: purified POMP91B protein. Lane M: Biorad molecular weight standard. (b) Motility tests of *E. coli* GI809 transformed with different constructions on IMC medium supplemented with tryptophan. Not transformed bacteria (A and D), bacteria transformed with the *p27* gene of *M. tuberculosis* (B) and with the *pomp91b* gene of *C. abortus* (C) inserted in the *FliCTrx* fusion gene. Swarming is observed after 28 hours incubation at 28 °C in a semi-gelosed medium containing 0.3% motility agar. (c) Western Blot showing the expression of hybrid flagellin without insert in the Flagellin-thioredoxin (Lane 1), or with the partial POMP91B inserted in the thioredoxin (Lane 2).

The partial POMP91B recombinant protein was expressed in *E. coli* BL21(DE3) and purified using Ni-gel chromatography. The purified POMP91B protein fragment was analysed by SDS-PAGE as shown in Figure 1(a). For the expression of POMP91B into the flagellin of *E. coli*, the 5’ part of the *pomp91B* gene was cloned in frame with the *fliC-trx* fusion gene present on the plasmid pFliTrx. The gene was inserted in the unique *Rsr*II site of the thioredoxin gene allowing directional insertion. Positive colonies were tested by PCR and confirmed by DNA sequencing. To control the expression of the new chimeric flagellin two tests were used. In the first test, cells were assayed by swarming test as described in the Experimental Section 4.2. Results in Figure 1(b) indicate that cells carrying the POMP91B hybrid flagellin were motile in semi-solid IMC medium supplemented with tryptophan. In the second test, tryptophan-induced cell lysates were fractionated on SDS gel, transferred to a membrane and probed with monoclonal anti- thioredoxin or anti-POMP91B antibodies, thus confirming the presence of POMP91B protein in transformed cells [Figure 1(c)]. 

### 2.2. Cellular immune response - Cell proliferation

**Figure 2:**
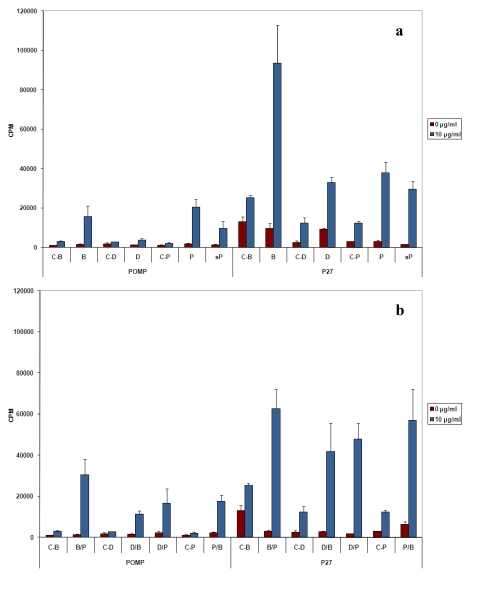
Proliferation of splenic cells of immunized mice. (a) Splenic cells from mice immunized either with flagellin modified bacteria (B), DNA plasmid containing the genes *pomp91b orp27*  of *M. tuberculosis* (D) or with the POMP91B or p27 recombinant proteins associated (P) or not (sP) with Freund's adjuvant, were incubated *in vitro* with or without 10 mg/mL of purified POMP91B or p27 recombinant protein. The proliferation was monitored by [^3^H] thymidine uptake (±standard errors) at 66 h after stimulation. Control groups have been immunized with non-modified bacteria (C-B), the empty pcDNA3 plasmid (C-D) or with PBS in Freund’s adjuvant (C-P). (b) Response of splenic cells from mice received combined immunizations which were indicated by two letters. B/P means that the first immunization was with flagellin modified bacteria (B) and the following immunization with the protein in Freund’s adjuvant (P); D/B, D/P: the first immunization was with DNA then by bacteria (D/B) or protein (D/P); and P/B, the first immunization was with the protein then by bacteria.

The cellular responses were first evaluated by measuring the proliferation of splenic cells after *in vitro* stimulation with the recombinant POMP91B protein. Proliferation was determined with a classical [^3^H] thymidine incorporation assay. Groups of mice were divided into a subset that was antigen-boosted one week before the test and another subset that was not boosted. No significant differences in the cellular responses were observed between the two subsets (data not shown). Bacteria with non-modified flagellin (C‑B), plasmid with no insert (C-D) and PBS (C-P) were used as negative controls, respectively.

Unlike P27 antigen that was able to induce strong cellular proliferations (Figure 2 and [20]), especially after immunization with the recombinant bacteria having the p27 protein inserted in flagellin, the POMP91B antigen was unable to promote such an intense response, regardless of the immunization procedure used. Immunization with the bacteria (group B) gave the highest proliferation (93.517 cpm ± 19.254) for P27 antigen, whereas only 15.592 cpm ± 5.383 was obtained when the POMP91B antigen was used [Figure 2(a)]. Immunization by the recombinant protein emulsified first in CFA then by IFA (group P) induced a small increase in proliferation (20.544 cpm ± 3.850), but the levels were inferior when compared to those observed with the P27 antigen (38.023 ± 5.223). The weakest responses were obtained by DNA immunizations using pcDNA3 plasmid containing the *pomp91B* gene (group D), *i.e.*, 3.795 cpm, or by the soluble protein without any adjuvant (group sP), *i.e.*, 9.728 cpm.

Combined immunizations with bacteria followed by boosts with the POMP91B protein in IFA (group B/P) as shown in Figure 2(b), increased the intensity of the response as compared to that obtained by the modified bacteria alone (15.592 cpm ± 5.383 to 30.533 cpm ± 7.574), and gave the strongest proliferation for this antigen, a result that is contrary to that observed with the P27 antigen (decrease from 93.517 cpm ± 19.254 to 62.593 cpm ± 9.590). In contrast to POMP91B, immunization with protein in CFA followed by immunization with the flagellin modified bacteria (group P/B) led to a relatively decreased response (20.544 cpm ± 3.850 to 17.693 cpm ± 3.001). DNA priming followed by the POMP91B protein (group D/P) or modified bacteria (group D/B) boosts also led to an increase in response intensity (3.795 cpm to 16.703 cpm and 11.347 cpm respectively).

### 2.3. Cellular immune response - Recombinant protein immunization induces a Th2-like type of immune response

**Figure 3:**
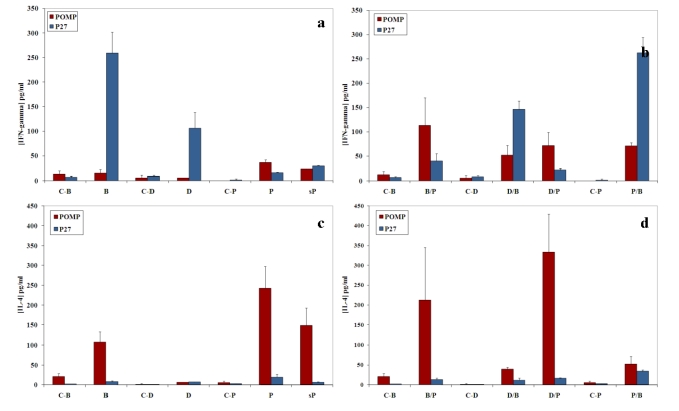
Cytokine secretion by splenic cells of immunized mice. Splenic cells were stimulated *in vitro* by the recombinant POMP91B and p27 protein and IFN-γ (a and b) and IL-4 (c and d) were quantified in the supernatant after one week of culture. Results are presented as mean cytokine concentrations (±standard errors) compared to a standard curve of purified cytokines as mentioned in materials and methods. Controls and immunization as in Figure 2 (a and c for homogenized immunization, b and d for combined immunization).

To investigate the cytokine profile induced by the different immunization methods (plasmid DNA, recombinant protein, bacteria with modified flagellin), we measured the amount of antigen-specific IFN‑γ and IL-4 secreted by splenic cells isolated from immunized mice. The spleens were harvested one week after the last injection. Cell suspensions were stimulated *in vitro* with the POMP91B or P27 recombinant protein using different concentrations. The cytokine tests were performed on the cells supernatants after 48 hours and after one week of culture. At 48 hours a weak response was observed for both cytokines (data not shown). However, one week later high levels of IL-4 were secreted by splenic cells from the group of mice immunized by the recombinant protein emulsified in Freund’s complete and incomplete adjuvant [Figure 3(c) and Figure 3(d); indeed, one of the three groups that secreted the highest IL-4 concentration was that of mice immunized with the plasmid and then recombinant protein (333 pg/mL)]. The two other groups boosted with the recombinant protein in IFA, B/P and P, also induced a high release of IL-4 (212 and 243 pg/mL respectively). The group immunized with the protein alone had IL-4 concentration of 149 pg/mL. In the same conditions, P27 was unable to promote such a production of IL-4, and this level remains low for all other tested groups [Figure 3(c) and Figure 3(d)]. Cells from mice immunized by bacteria carrying the antigen POMP91B in their flagellin (B, D/B and P/B) produced lowest amounts of IL-4 (107, 39 and 52 pg/mL, respectively) whereas IL-4 released by cells from mice immunized exclusively with plasmid DNA (6.5 pg/mL) is not significantly different from the control.

IFN‑γ production [Figure 3(a) and Figure 3(b)] for the POMP91B antigen was low in respect to that obtained with the P27 antigen. The highest response (113 pg/mL) was obtained with cells from mice primed with flagellin-modified bacteria and boosted with recombinant protein in IFA (B/P group). All other groups (P, sP, D/B, D/P, P/B) showed comparable IFN-γ levels, *i.e.*, between 26 and 72 pg/mL, with the exception of the groups B and D in which IFN-γ release was completely absent. This is opposite to the effect of P27 antigen since the B group induced the best response [Figure 3(a)].

### 2.4. Antibody responses

To investigate the anti-POMP91B antibodies titer in the sera of mice immunized with different combinations of antigens and their controls, the levels of IgM, IgG1, IgG2a, IgG2b, IgG3 and IgA were determined by ELISA one week after the second and the third immunizations. The optical densities (ODs) obtained for the four IgG isotypes, IgA, and IgM, were measured with sera diluted at 1/500.

**Figure 4:**
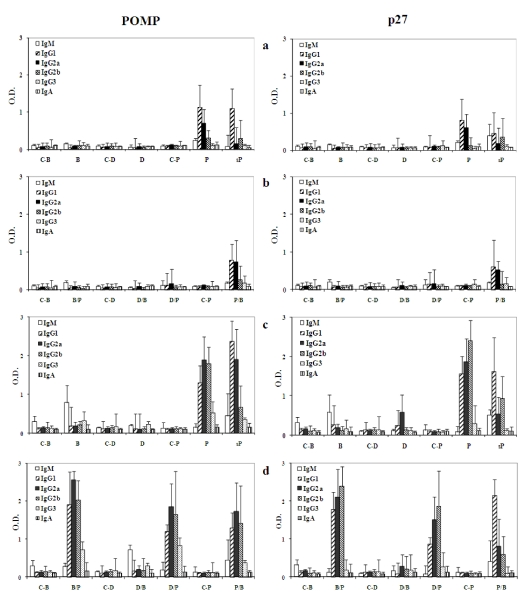
Specific anti-POMP91b and anti-p27 antibodies responses. Mice sera diluted at 1/500 were tested in ELISA for the presence of anti-POMP91B or anti-p27 antibodies of the different isotypes IgG1, IgG2a, IgG2b, IgG3, IgM and IgA one week after the second (a and b) or the third (c and d) immunization. The results are presented as the optical density (±standard errors) of the different isotypes. Histograms a and c are for groups with homogenized immunization, b and d for groups with combined immunization. Controls and immunizations as in Figure 2.

Similar to the cellular response, the antibody response depended on the PAMPs used during immunization. After the second (first boost) immunizations [Figure 4(a)], the group immunized with the protein in Freund’s adjuvant (P) gave the strongest antibody response; for IgG1 (O.D. = 1.130) and for IgG2a (O.D. = 0.702). Without Freund’s adjuvant, when the protein was in saline solution (sP), the response was also robust, but only for IgG1 (O.D. = 1.101) and not for IgG2a (O.D. = 0.156).

The group immunized with flagellin modified bacteria (B) only developed a weak IgM response (O.D. = 0.153), and the DNA immunized group did not show any significant antibody response. The response obtained by group (P/B) [Figure 4(b)], primed with recombinant protein in CFA and boosted by the flagellin-modified bacteria showed weaker IgG2a response as compared to that observed for the Freund’s adjuvant group (O.D. = 0.788 and O.D. = 0.748 for IgG1 and IgG2a respectively). No significant responses were noted for the other groups, except for a weak IgM response in the two groups primed with the modified bacteria and B/P (O.D. = 0.153 and O.D. = 0.187 respectively). 

After the third immunization [Figure 4(c) and Figure 4(d)], stronger responses were observed in all immunized groups. The best response was obtained in mice primed with flagellin-modified bacteria and boosted with the protein emulsified in incomplete Freund’s adjuvant (B/P) [Figure 4(d)], i.e., O.D. = 2.554 for IgG2a, 2.018 for IgG2b and 1.903 for IgG1. Three other groups (P, D/P and P/B) present similarly weaker antibody isotype response with the highest response with IgG2a, followed by IgG2b and then IgG1. The order of intensity was different with the P27 antigen since the IgG2b was dominant, followed by IgG2a and then by IgG1. On the contrary to the P27 antigen, in these four groups POMP91B promoted IgG3 responses. In the group primed and boosted with the flagellin-modified bacteria (B), IgM was the only significant immunoglobulin (O.D. = 0.780). This is similar to the group boosted by the flagellin-modified bacteria after priming with DNA (D/B) (O.D. = 0.722). No significant augmentation of any isotype could be detected in sera from mice immunized with the DNA plasmid for the POMP91B antigen, in contrast to the effect of P27 where IgG2a could be observed. Mice in the sP group gave a strong IgG1 response (O.D. = 2.370) [Figure 4(c)] but has also a good IgG2a response (O.D. = 1.905) that is not seen with the P27 antigen. IgA responses were negligible regardless of the nature of the antigen used for immunization.

## 3. Discussion

Two conclusions may be drawn from our results; first the difference between the two immune responses (*i.e.*, Th1 *vs*. Th2) is determined by the nature of antigen, and the second concerns the difficulty in obtaining a cellular immune response against the POMP91B antigen of the obligateintracellular growth pathogen *Chlamydophila abortus*.

Using different PAMPs including bacterial flagellin which is recognized by TLR5, Freund’s adjuvant which contains muramyl dipeptides and lipoarabinomannans, the ligand of the TLR2, and the CpG DNA recognized by the TLR9, we studied the immune response against the *N*-terminal part of the POMP91B protein of *Chlamydophila abortus*,which was found to be strongly immunogenic [7]. Then we compared them with those obtained in the same conditions with the p27 PPE antigen, a cell surface protein from *M. tuberculosis* [20]. Unlike the p27 PPE antigen that induced a strong proliferative response accompanied by high production of IFN‑γ and low amount of IL-4, the POMP91B antigen induced in general a Th2-like response, with weak proliferation of cells, low production of IFN-γ and high levels of IL-4. This is particularly true when immunizations are practised with the recombinant protein (Figure 2 and Figure 3). The results indicated that this antigen may be involved in the establishment of the host humoral immune response against *C. abortus*. However, variations were observed in the intensity of the responses depending on the method of immunization used. While with p27 the strongest proliferative response of splenic cells in our experimental conditions was obtained utilizing the flagellin immunization, POMP91B antigen was not able to induce such a strong response and a better response was only obtained by immunization with the recombinant POMP91B antigen with incomplete Freund’s adjuvant after priming with flagellin-modified bacteria. In addition, the highest amount of IFN‑γ production was found in cells derived from mice immunized using the same combination. It is interesting to note that for POMP91B, as for p27, the group that showed the best cell proliferation was also producing the highest amount of IFN-γ. IFN‑γ is one of the cytokine known to be important in promoting a Th1 response and enhancing cytotoxicity during the priming and maturation of lymphocytes [22]. 

In our experiments, the proliferation response induced by DNA immunization was the lowest and the production of IFN‑γ was not significantly different from the control. The pcDNA3 plasmid used as the immunization vector contains two GACpGTT motifs, which are known to induce Th1 cytokines in mice [23,24]. However, a synergistic effect was observed between DNA vaccination and flagellin expression vector [25].

The results with POMP91B are also different from our previous observation concerning the immune response to *Clostridium tyrobutyricum* flagellin, in which a good cellular and antibody response were obtained using the flagellin as carrier and adjuvant [19]. The absence of IFN-γ production using the POMP91B antigen and the dominance of Th2 like response were also observed with another antigen, the Major Outer Membrane Protein (MOMP) of *C. trachomatis* using another immunization system in which murine dentritic cells were pulsed with the antigen [26]. These observations showing that surface proteins of *Chlamydia* genus do not seem to induce a CD4+ Type1 response could be the reason for the failure to produce the needed vaccine to prevent infections. It is evident that IFN-γ is an important effector cytokine, particularly in the clearance of infections caused by human strains [27,28].

Despite the absence of a strong cellular response with POMP91B antigen, the role of flagellin in the induction of cellular responses could be clearly demonstrated by the use of combined immunizations. Cells isolated from mice primed by the antigen presented on the modified flagellin and boosted with protein in IFA released the highest amount of IFN‑γ [Figure 3(b)]. 

In conclusion, we found that the administration of recombinant bacteria carrying the P27 PPE *M. tuberculosis* antigen resulted in the induction of Th1 response. This kind of response may play a key role in protection against intracellular pathogens. Unfortunately, this immunization did not give such a good response with the POMP91B *C. abortus* antigen; however, both are cell surface exposed bacterial antigens. These results suggest that the nature of the antigen could influence the Th1-Th2 balance of the immune response. Only the three-dimensional structure of the extracellular *N*‑terminal part of POMP91B was determined and it represented a compact beta-helical domain with six rungs [11]. Combined immunization can be useful to obtain a global response (antibody and cytokine production). Indeed using POMP91B antigen, the best global response was observed in the group primed by flagellin-modified bacteria that was then boosted by the recombinant protein in incomplete Freund’s adjuvant [Figure 2(b), Figure 3(b), Figure 3(d) and Figure 4(d)]. 

Concerning the humoral response to POMP91B in recombinant protein form with Freund’s adjuvant, the fact that the major antibody isotype primed is IgG2a, is quite surprising since this isotype is promoted by a Th1-like response. However, when the POMP91B antigen is injected in saline (sP) without any adjuvant, the IgG1 isotype of the Th2-like response is the one showing the strongest intensity. Here again, the study of the antibody responses confirmed our previous observations showing that the flagellin and the DNA system of immunization were unable to induce a good antibody response. In contrast, the optimal response resulted from the classical method using Freund’s adjuvant. For the flagellin immunization, the major isotype found in the sera was the IgM. The interaction of flagellin with the immune cells in our experiments failed to lead to an isotype switch.

## 4. Experimental Section

### 4.1. Bacterial strains, plasmid and antigens preparation

*Escherichia coli* strains DH5a (Invitrogen, San Diego, CA, USA) and BL21 (DE3) (Novagen, San Diego, CA, USA) were used for cloning and overexpression of the recombinant protein, respectively. For the production of recombinant protein, the 5’part of the *pomp91B* gene was amplified by PCR from an original template plasmid provided by Dr. Olivier Grépinet (INRA Tours, Nouzilly, France) using oligonucleotide pair 5’-GGG AAT TCC ATA TGA AAC ATC CAG TCT ACT GGT T-3’ and 5’-CGC GGA TCC GGA CGG TGT TTG TAA CGT A-3’. The oligonucleotide primers (Eurogentec, Seraing, Belgium) contained a *Nde*I and a *Bam*HI site (underlined) respectively. PCR products were purified, digested by the two restriction enzymes *Nde*I and *Bam*HI and ligated during 16 h at 4 °C with T4 ligase (Eurogentec) into the expression vector pET15b (Novagen) pre-digested with *Nde*I and *Bam*HI. This plasmid encodes six histidines tagged to the *N*-terminus of the expressed recombinant protein to be purified. Expression of the recombinant protein was induced by adding 1 mM isopropyl-*b*-_D_-thiogalactopyranoside (IPTG) to BL21(DE3) bacteria (OD_600_ = 0.6) carrying the recombinant pET-15b-*pomp91b* plasmid in 2 × TY medium at 28 °C for 3 to 4 hours. The bacteria were harvested by centrifugation, resuspended and lysed by incubation for 1 hour in lysis buffer (100 mM Na_2_HPO_4_, 10 mM Tris-HCl, 8 M urea, pH 8.0). After centrifugation (10,000 g, 30 min), the supernatant was added to a Ni-NTA resin (Qiagen, Hilden, Germany). The mixture was incubated for 1 h at room temperature. After two washes with the same buffer, recombinant proteins were eluted with urea 8 M at pH of 5.9, then 4.5. The purified recombinant proteins were renatured by dialysis against PBS pH 7.4 at 4 °C, quantified by a Bradford assay, filtered, and stored in the same buffer at ‑20 °C.

For DNA immunization, the gene encoding the N-part of the POMP91B protein was cloned in the eukaryotic expression vector pcDNA3 (Invitrogen). Plasmid DNA was amplified in *E. coli* DH5a, purified by Qiagen purification kit, dissolved in phosphate-buffered saline (PBS), and stored at ‑20 °C until use. 

For immunizations with the bacteria carrying the POMP91B protein in their flagellin FliC protein, a plasmid (pFliTrx) coding for *fliC* gene and a specific *E. coli* flagellin-deficient strain were used as described [21]. The plasmid pFliTrx, carries the incomplete *E. coli fliC* coding sequence in which the coding region for amino-acid residues 244-351 of the *E. coli* flagellin has been replaced, in frame, with the entire coding sequence for the *E. coli* thioredoxin (*trxA*) gene. The partial *pomp91b* gene was cloned in the *trxA* gene at the unique *Rsr*II restriction site. The two primers used (5’-GGG GGG GTC CGA AAC ATC CAG TCT ACT GGT-3’ and 5’-GGG GGG GAC CGG ACG GTG TTT GTA ACG TA-3’) have an *Ava*II restriction site (underlined) allowing a directional cloning. Plasmid constructs were then used to transform the non-motile *E. coli* GI809 strain [21], which carries a specific 512 bp deletion within the flagellin gene (*fli*C, GenBank accession #M14358). Transformed motile bacteria were assessed for motility by swarming assay and by western blotting using specific antibodies, before preparation of immunization solutions.

### 4.2. Swarming assays

Non-motile *E. coli* GI809 cells were transformed by a flagellin containing pFliTrx plasmid and placed in a Petri dish containing IMC semi-gelosed medium with an agar specific for motility (Difco, Detroit, MI, USA) at 0.3%. The incubation was realized at 28 °C and the motility was observed at least 15 h after the start of the culture.

### 4.3. SDS-PAGE and western blot analysis

Purified proteins and bacterial extracts were heated 5 min at 100 °C in the lysis buffer (50 mM Tris, pH 8.0, 150 mM NaCl, 4% SDS, 5% b-mercaptoethanol) and electrophoresed through a 12.5% polyacrylamide gel, transferred to nitrocellulose membranes, then blotted with anti-POMP91B or anti-thioredoxin antibodies as described in Le Moigne *et al*. [19].

### 4.4. Animals and immunization

Adult female BALB/c mice (Janvier, Le Genest, France) were divided in groups of six mice and immunized either with 50 mg of plasmid DNA carrying the antigen coding sequence, in 100 mL PBS, three times with one month interval in the quadriceps muscle (DNA immunization), with 25 mg of antigen emulsified in complete Freund's adjuvant (CFA, containing the H37Ra, ATCC 25177 *M. tuberculosis* strain) for the first immunization or incomplete Freund's adjuvant (IFA) for the two following immunizations, by a subcutaneous route (Classical immunization), with 50 mg of antigen in PBS (Soluble antigen) or with 10^8^ modified *E. coli* bacteria which express the antigen into the flagellin at the surface of the bacteria given intraperitonneally (Flagellin immunization). Control mice for DNA immunization were immunized with empty pcDNA3 plasmid, whereas control mice for classical immunization and soluble antigen immunization were immunized with PBS incorporated in Freund's adjuvant or PBS alone, respectively. For the flagellin immunization, control mice were immunized with non modified *E. coli* (bacteria which express normal flagellin). Combined immunizations between the different methods were applied as in Table 1. 

**Table 1:** Group’s name in function of the different immunization practiced *.

		Method of immunization						
		Classical Immunization.		DNA Immunization			Flagellin Immunization	
First immunization	Soluble protein	Protein + CFA		Eukaryotic plasmid			Modified bacteria	
Following Immunizations	Soluble protein	Protein + IFA	Bacteria	Protein + IFA	Bacteria	Plasmid	Protein + IFA	Bacteria
Group’s name	sP	P/P	P/B	D/P	D/B	D/D	B/P	B/B

* Control animals have received PBS or no related protein mixed with CFA for classical immunization (C/P), empty pcDNA3 or pcDNA3 containing no related gene for DNA immunization (C/D) and normal *E. coli* or *E. coli* expressing no related proteins in their flagellin for bacterial immunization (C/B).

Blood samples were collected one week after the last immunization from the retro‑orbital plexus and sera were stored at −20 °C until use. All animal experiments were done in accordance with institutional and national ethical guidelines.

### 4.5. Enzyme-linked immunosorbent assay (ELISA)

Plates coated overnight at 4 °C with 1 µg/mL of recombinant proteins in 100 µL of carbonate-bicarbonate buffer (0.1 M, pH 9.6), were washed with phosphate-buffered saline-Tween 20 (PBS-T) (0.05%; v/v) and blocked for one hour at 37 °C with PBS-T containing 0.5% gelatin (PBS-T-G). Then serum dilutions (1/500) in the same buffer were added. After 1 h of incubation at 37 °C and four washes, goat anti-mouse IgM, IgG1, IgG2a, IgG2b, IgG3 and IgA alkaline phosphatase-conjugate (Southern biotechnology, Birmingham, AL, USA) were added and the plates were incubated for another 1 h at 37 °C. After four washes, 100 µL of 1 mg/mL of *p*-nitrophenylphosphate (Sigma, Saint Quentin Fallavier, France) in diethanolamine buffer (pH 9.8) was added, and then plates were read at 405 nm with a Titertek Multiscan instrument (Skatron, Oslo, Norway). 

### 4.6. Proliferation assay

To check the effect of antigen boosting in our system, only half of the mice were boosted intraperitonneally with 25 mg of soluble antigen in PBS six months after the first immunization. One week later, splenic cells (5 × 10^5^ cells/well) from antigen-boosted or non-boosted mice were suspended in RPMI-10% (FCS) and plated in 96-well flat bottomed plates containing serial dilution of antigen. Two days later, 0.5 µCi of [^3^H] thymidine was added to each well and incubated for an additional 18 hours, then cells were harvested and thymidine incorporation was measured using a liquid scintillation spectrophotometer Packard 1600 Tricarb (Packard, Downers Grove, IL, USA).

### 4.7. Cytokine production and quantification by ELISA

Splenic cells (5 × 10^5^ cells) from boosted, non-boosted and control mice were cultured in RPMI-10% fetal calf serum (FCS) at 37 °C in 5% CO2 in 96-well plates containing different concentrations: 0, 1.1, 3.3 and 10 µg of recombinant proteins per mL. Supernatants were harvested after 48 h and one week of incubation and stored at ‑20 °C until use. IL-4 and IFN‑γ contents were measured with a commercial ELISA kit (Bender MedSystems, Vienna, Austria) according to the manufacturer’s protocol.

## 5. Conclusions

Our results indicate that the protective immune response to an antigen depends on many factors including the vector, the adjuvant and the nature of the antigen. One or more of these elements may work for large spectre of antigens but not for all. In this case, different ways of immunizations should be combined to obtain a good immune response.
